# Usefulness of Proguanylin, Pentraxin 3 and S100A12 Serum Concentrations in Diagnosis and Monitoring the Disease Activity in Crohn’s Disease

**DOI:** 10.3390/biom13101448

**Published:** 2023-09-26

**Authors:** Aleksandra Kałużna, Agnieszka Jura-Półtorak, Alicja Derkacz, Krystyna Olczyk, Katarzyna Komosinska-Vassev

**Affiliations:** 1 Department of Clinical Chemistry and Laboratory Diagnostics, Faculty of Pharmaceutical Sciences in Sosnowiec, Medical University of Silesia in Katowice, 41-200 Sosnowiec, Poland; ajura@sum.edu.pl (A.J.-P.); olczyk@sum.edu.pl (K.O.); kvassev@sum.edu.pl (K.K.-V.); 2 City Hospitals of Chorzów, 41-500 Chorzów, Poland; derkacz.alicja@gmail.com

**Keywords:** proguanylin, PTX3 protein, S100A12 protein, Crohn disease, colitis, ulcerative, intestinal mucosa, inflammation, neutrophils

## Abstract

The aim of our case-control study was to identify novel biomarkers of Crohn’s disease (CD) that hold the potential to be employed in both disease diagnosis and monitoring activity. In the context of the contribution of intestinal barrier integrity and immune response to the pathogenesis of CD, we assessed the serum concentrations of proguanylin (pro-GN), pentraxin 3 (PTX3) and S100A12 in 20 patients before and after anti-inflammatory treatment, as well as in 20 healthy individuals. Statistical analyses revealed a significant difference in the levels of pro-GN (5.5 vs. 11.35, *p* < 0.001), PTX3 (2117.9 vs. 1608.37, *p* < 0.05) and S100A12 (79.4 vs. 19.74, *p* < 0.001) between pretreatment patients with CD and healthy individuals. Moreover, we noted a significant relationship between the serum profile of PTX3 and disease activity, expressed as CDAI, both before (*p* < 0.005, r = 0.63) and after (*p* < 0.05, r = 0.60) treatment. A similar correlation was noted in the case of S100A12 (*p* < 0.005, r = 0.81), albeit exclusively within the post-treatment group of patients. Anti-inflammatory treatment resulted in an elevation of pro-GN concentration (5.5 vs. 8.04, *p* < 0.001) and a reduction in PTX3 level (2117.9 vs. 1609.5, *p* < 0.05) in the serum of patients with CD. In comparison to our previous research conducted on a group of patients with ulcerative colitis (UC), those with CD exhibited reduced levels of PTX3 (2117.9 vs. 3197.05, *p* < 0.005) and elevated concentrations of S100A12 (79.4 vs. 39.36, *p* < 0.05). The results obtained from this investigation suggest that measurements of pro-GN, PTX3 and S100A12 could prove beneficial in the diagnosis of Crohn’s disease. Assessment of changes in the serum profile of PTX3 appears to be a good marker of response to treatment but also, along with analysis of S100A12 protein serum levels, a useful marker in differentiating CD from UC.

## 1. Introduction

Inflammatory bowel disease (IBD) is a chronic and inflammatory condition characterized by alternating episodes of clinical remission and relapse. It can be classified into two primary types of disorders—ulcerative colitis (UC) and Crohn’s disease (CD). In UC, the disease spreads proximally from the colon to other segments of intestines, affecting the mucosa and submucosa layers. In contrast, CD can lead to lesions anywhere along the gastrointestinal tract, extending through the entire tissue [1,2]. The onset of Crohn’s disease is particularly problematic, as half of the patients experience intestinal complications such as strictures or fistula. Additionally, 43% of patients present extraintestinal symptoms like psoriasis or arthritis. Moreover, patients with CD are burdened with increased risk of colorectal and small-bowel cancers. Therefore, regarding the severe course of the disease and its possible complications, early diagnosis of Crohn’s disease is extremely important [1]. Nevertheless, due to the challenges associated with differential diagnosis as well as the divergent phenotypes of CD, ranging from penetrating to sctricturing types, the diagnosis may be significantly delayed and may even exceed 2 years. The current gold standard for diagnosing CD involves endoscopic examination, which is a valuable but highly invasive method. At the same time, noninvasive methods that could support the diagnostic process remain limited and possess low specificity. Therefore, numerous studies are dedicated not only to examining new diagnostic methods but also to correlating serological or fecal biomarkers with the clinical status of patients with Crohn’s disease. Consequently, the identification of new biomarkers that could aid in both the diagnosis and monitoring of the disease’s progression is of particular interest to researchers [1,3,4,5,6].

The pathogenesis of Crohn’s disease involves multiple factors, but a crucial role is contributed to impaired intestinal barrier integrity and exceeded immune response [1]. In our research to identify new potential biomarkers of Crohn’s disease, we focused on parameters related to intestinal barrier integrity or to immune cells involved in the initiation and progression of the disease. The first analyzed biomarker is proguanylin (pro-GN), which serves as the most prevalent circulating precursor of guanylin. Guanylin acts as an endogenous agonist of guanylyl cyclase C (GC-C), a receptor located on the surface of intestinal epithelial cells (IECs). Upon cleavage of proguanylin, guanylin is absorbed from the lumen into intestinal mucosa, where it locally activates the GC-C signaling pathway [2,7]. The mentioned pathway plays a crucial role in maintaining the hydro-electrolyte balance, and its activation inhibits the absorption of sodium ions by IECs while enhancing the secretion of chloride ions, resulting in water efflux. Considering that hydro-electrolyte homeostasis plays a vital role in the proper functioning of the intestinal barrier, guanylin not only regulates the ionic and fluid balance but also may be associated with preserving the functionality of the intestinal barrier. Moreover, it is suggested that the GC-C pathway might protect the intestinal barrier integrity by regulating the expression of tight junction proteins (TJPs), promoting the proliferation of IECs and facilitating the proper synthesis of mucus, which serves a protective role in maintaining the intestinal epithelium [2,7,8]. Considering the potential role of guanylin in maintaining the intestinal barrier integrity and the fact that proguanylin is the most prevalent precursor of GC-C agonist in circulation, the measurements of pro-GN in the serum of patients with Crohn’s disease may be useful in the diagnosis and monitoring of the activity of the disease.

Other potential biomarkers of Crohn’s disease analyzed in this study are pentraxin 3 (PTX3) and s100A12 protein, both of whose function is related to the immune response. PTX3 is a glycoprotein belonging to the pentraxin family along with the well-known C-reactive protein (CRP). Similar to CRP, PTX3 plays a role in innate immune response, and its expression increases under inflammatory conditions. The synthesis of PTX3 occurs in various cell types, including monocytes/macrophages, epithelial cells, endothelial cells and smooth muscle cells. Neutrophils also serve as a cellular source of PTX3, storing the protein in their granules and releasing it immediately upon appropriate stimuli. The stimuli controlling synthesis and release of PTX3 include inflammatory cytokines, including IL-1β, TNF-α and microbial antigens like lipopolysaccharide (LPS) [9,10]. In the context of CD, impaired intestinal barrier integrity can lead to the exposure of IECs to microbial antigens, including LPS, thereby triggering the activation of innate immune cells and their migration to the inflammatory site. Neutrophils are among the first cells to infiltrate the intestinal mucosa, and upon activation, they can form neutrophil extracellular traps (NETs) to immobilize and neutralize pathogens. This activation of neutrophils and subsequent NET formation result in the release of approximately 25% of stored PTX3, which can opsonize pathogens, regulate complement activation and modulate the cytokine expression [1,9,10]. Given the aforementioned role, PTX3 may not only be upregulated during Crohn’s disease but also play a crucial role in the development of the disease. Another neutrophil-derived inflammatory product, S100A12, may similarly contribute to the initiation and progression of Crohn’s disease. The mentioned protein belongs to the same family as calprotectin, a parameter commonly assessed in the diagnosis of gastrointestinal diseases. S100A12 plays an antimicrobial role and is involved in the regulation of innate immunity, acting as a ligand for the receptor for advanced glycation products (RAGE). The binding of S100A12 to RAGE leads to the activation of pro-inflammatory nuclear factor κ B (NF-κB) and mitogen-activated protein kinase pathways, followed by the secretion of cytokines and adhesion molecules that induce the inflammatory process and enhance the migration of immune cells to the inflammatory site [11,12]. The release of cytokines (IL-6, TNF-α) induces the synthesis and secretion of S100A12 from another cellular source—monocytes/macrophages. This, together with the intensified influx and activation of neutrophils, can significantly increase the concentration of S100A12. An elevated level of S100A12 can bind to RAGE, leading to further upregulation of the NF-κB pathway and creating a vicious cycle of inflammation [12,13]. In Crohn’s disease, enhanced expression of both RAGE and S100A12 has been observed. Moreover, in biopsies collected from IBD patients, the level of RAGE was correlated with the inflammatory status, highlighting a crucial role of this receptor in the progression of the disease [13]. Therefore, an increased level of S100A12 may reflect disease activity as well as the intensity of the inflammatory process.

Regarding the contribution of proguanylin to intestinal barrier integrity and pentraxin 3 along with S100A12 to innate immunity, these mentioned biomarkers may be useful in the diagnosis of Crohn’s disease. Moreover, as the analyzed biomarkers are related to the inflammatory response, playing a key role in the progression of the disease, measurement of pro-GN, PTX3 and S100A12 might also be valuable in assessing disease activity and the intensity of the inflammatory process. Therefore, the hypothesis of our study is that pro-GN, PTX3 and S100A12 measurements in the serum of patients with Crohn’s disease are useful in diagnosing and monitoring the activity of the disease.

## 2. Materials and Methods

### 2.1. Study Population and Study Design

The material investigated in this research consisted of venous blood collected from 40 adults including 20 healthy individuals and 20 patients with Crohn’s disease before treatment and after a year of anti-inflammatory therapy. The exclusion criteria for the control group were hospitalization or surgery within the year prior to study as well as pharmacological treatment in the prior month. Additionally, all the laboratory parameters assessed in the blood of healthy individuals had to be within the reference ranges. For the study group, the diagnosis of Crohn’s disease was made on the basis of clinical symptoms, endoscopic examination and laboratory tests at the Department of Gastroenterology of St. Barbara’s Regional Specialist Hospital in Sosnowiec. Moreover, the disease activity was evaluated using the Crohn’s Disease Activity Index (CDAI). The exclusion criteria among patients with CD were unstable coronary disease, myocardial failure, pregnancy or breastfeeding, diabetes, liver or kidney disease, severe viral, fungal or bacterial infections and precancerous or cancerous conditions. Patients included in this study were treated with prednisone, the dosage of which was adjusted for each patient individually and was gradually reduced up to 12 weeks. The anti-inflammatory treatment was further continued with azathioprine or mercaptiopurine.

This research is a case-control observational study in which we compared two groups: a control group comprising healthy individuals and a study group including patients with a prior diagnosis of Crohn’s disease. The aim of this study was to identify the significance of changes in the levels of pro-GN, PTX3 and S100A12 in the serum of the subjects depending on the presence or absence of the disease. In addition, our research was designed as a prospective study; therefore, we continued the observation of the study groups in order to determine the progression of the disease during treatment with corticosteroids as well as to identify changes in the levels of analyzed biomarkers after one year of the mentioned therapy. The study size was determined based on the aforementioned inclusion criteria, and subjects included in the research were only newly diagnosed patients with no prior corticosteroid treatment.

### 2.2. Assessing the Serum Proguanylin Concentration

Serum proguanylin level was measured using the Human Proguanylin ELISA test from BioVendor Company (Karasek, Czech Republic). The analytical sensitivity of the test was 0.06 ng/mL, while the intra-essay variability was estimated at the level of 5.2%.

### 2.3. Assessing the Serum Pentraxin 3 Concentration

Concentration of serum pentraxin 3 was assessed using a Human Pentraxin 3 ELISA test from BioVendor Company (Karasek, Czech Republic). The analytical sensitivity of the test equaled 22 pg/mL, while the intra-essay error was 3.4%.

### 2.4. Assessing the Serum S100A12 Concentration

Assessment of the serum S100A12 concentration was determined with the use of a Human S100A12 ELISA test from BioVendor Company (Karasek, Czech Republic). The analytical sensitivity of the method was estimated at the level of 0.02 ng/mL, and the intra-essay variability was 3.6%.

### 2.5. Statistical Analyses

Obtained data were characterized and evaluated with the use of STATISTICA software (StatSoft 13.3, Kraków, Poland). The normality of data distribution was assessed using the Shapiro–Wilk test. In the case of normally distributed data, the descriptive statistics included mean values and standard deviation (SD), while not normally distributed data were characterized by the median (Me) and interquartile range—lower (Q1) and upper (Q3) quartiles. The statistical significance of differences between patients with Crohn’s disease and healthy individuals was assessed with the use of Student’s *t* test in normally distributed data and the Mann–Whitney U test in not normally distributed data. Comparison of results before and after the implemented treatment was conducted using Student’s t test and the Wilcoxon test. The correlation between the concentration of analyzed biomarkers and disease activity along with CRP were evaluated using the Pearson test. In all tests and statistical analyses, a significance level of *p* < 0.05 was used.

## 3. Results

### 3.1. Research Data

**Figure 1:**
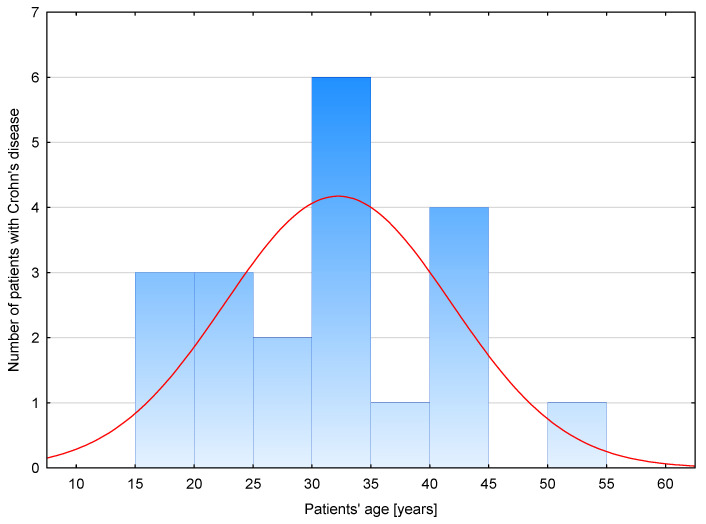
Age distribution in group of patients with Crohn’s disease.

The clinical characteristics of patients with Crohn’s disease are presented in Figure 1 and Table 1. The mentioned data were previously published, as part of the study group was previously included in a different study conducted at the Department of Clinical Chemistry and Laboratory Diagnostics of Medical University of Silesia [14]. In our research, we use Montreal classification to characterize patients according to disease location. The L1 (ileal) category was observed in the case of four patients, L2 (colonic) in seven patients, L3 (ileocolonic) in seven patients and L4 (isolated upper disease) in one patient. Due to anemia being a common complication of Crohn’s disease, we analyzed the concentration of hemoglobin as well as other parameters related to red blood cells. Before treatment, 55% of patients (11) had decreased level of hemoglobin, indicating anemia. Among those with anemia, 25% (five patients) presented an increased red blood cell distribution width (RDW-CV) and decreased mean corpuscular volume (MCV), suggesting microcytic anemia, which is one of the most frequent types of anemia in IBD. However, implemented anti-inflammatory treatment did not significantly affect the value of the mentioned parameters. Nevertheless, statistical analyses indicated a significant decrease in disease activity after the implemented treatment. Before anti-inflammatory therapy, the activity of disease was classified as moderate in 95% of patients and severe in 5%. After treatment, mild disease was diagnosed in 12.5% of patients, while 87.5% of patients presented moderate disease. To determine the effect of treatment on the inflammatory process, we measured the concentration of C-reactive protein (CRP) in the blood of patients with Crohn’s disease before and after therapy. Increased levels of CRP (≥5 mg/L) were noted in 14 patients in the pretreatment group and 12 in the post-treatment group [15,16]; however, conducted analyses revealed no significant change in CRP concentration after the implemented treatment.

### 3.2. Serum Profiles of Pro-GN, PTX3 and S100A12 in Patients with Crohn’s Disease and Healthy Individuals

**Figure 2:**
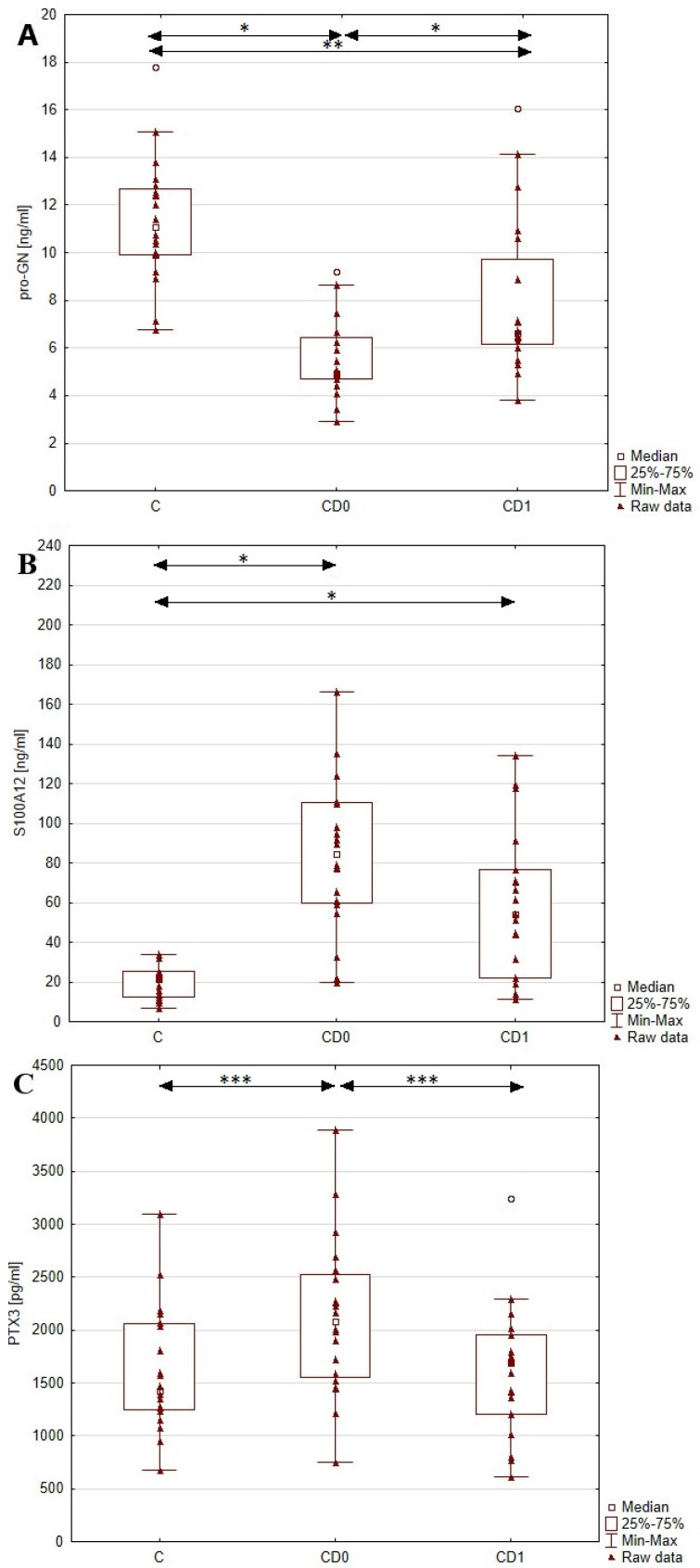
Comparison of serum profiles of pro−GN (**A**), S100A12 (**B**) and PTX3 (**C**) in healthy individuals and patients with Crohn’s disease before and after anti−inflammatory treatment. *, *p* < 0.001; **, *p* < 0.005; ***, *p* < 0.05; ° outliers; C, control group; CD0, patients with Crohn’s disease before treatment; CD1, patients with Crohn’s disease after treatment.

In this study, we aimed to evaluate the utility of pro-GN, PTX3 and S100A12 as diagnostic markers of Crohn’s disease. To achieve this, we assessed the levels of these mentioned parameters in the serum of patients with CD and healthy individuals, and the results are presented in Figure 2 and Table 2. The conducted statistical analyses revealed a significant difference in the serum concentrations of pro-GN, PTX3 and S100A12 between the pretreatment CD and control groups. The level of pro-GN in patients with Crohn’s disease was decreased (*p* < 0.001) by 52% compared to healthy individuals. At the same time, we noted a significant increase in PTX3 (*p* < 0.05) and S100A12 (*p* < 0.001) levels in the CD group by 32% and 302%, respectively, compared to control group.

### 3.3. The Relationship between Serum Levels of Pro-GN, PTX3 and S100A12 and Disease Activity along with Inflammatory State in Patients with Crohn’s Disease

**Figure 3:**
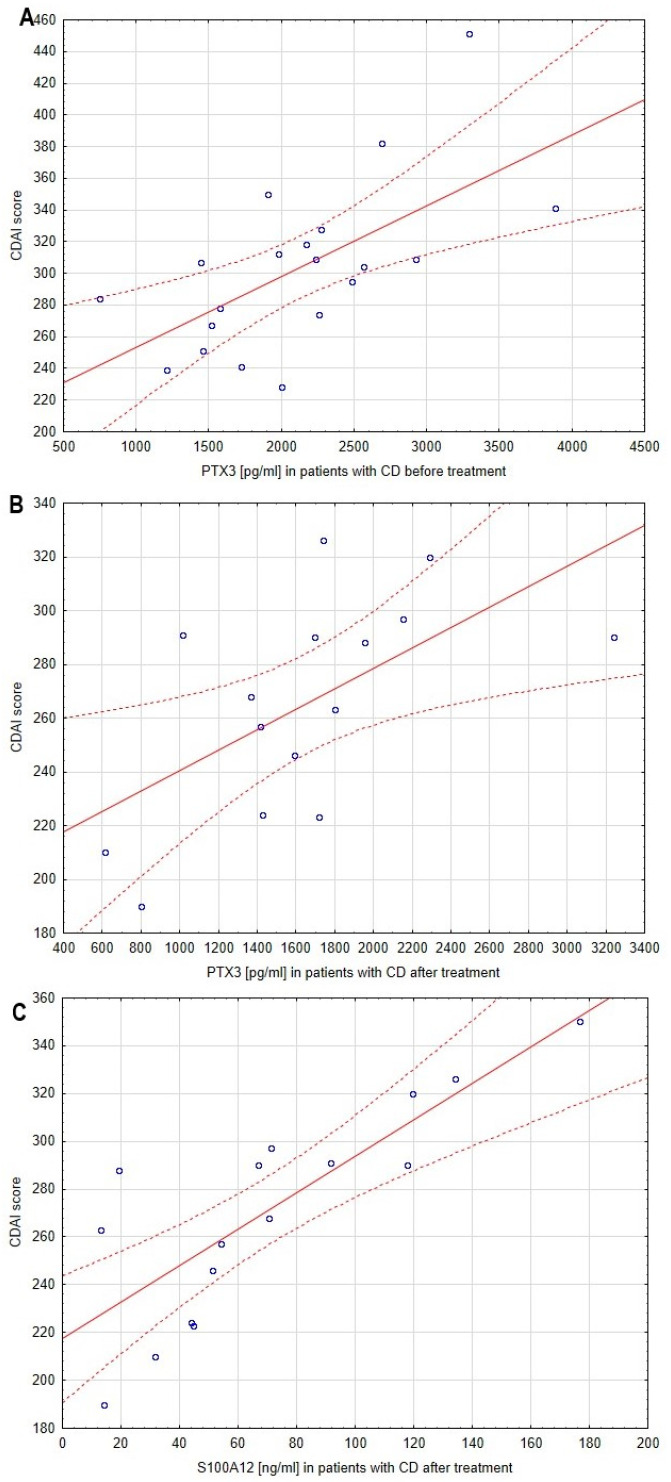
Correlations between serum profiles of analyzed biomarkers and disease activity in patients with Crohn’s disease. The correlations presented in this figure are exclusively data with statistical significance. (**A**), correlation between CDAI score and PTX3 serum profile in patients with CD before treatment; (**B**), correlation between CDAI score and PTX3 serum profile in patients with CD after treatment; (**C**), correlation between CDAI score and S100A12 serum profile in patients with CD after treatment; solid line, regression line; dotted line, 95% confidence interval; °, values noted in individual patient.

Regarding the aim of this study to identify new biomarkers reflecting disease activity, we evaluated the correlation between serum profiles of pro-GN, PTX3 and S100A12 and the CDAI score. The correlations between CDAI score and the analyzed biomarkers are presented in Table 3 and Figure 3. No significant relationship was noted between the CDAI score and pro-GN serum concentration before as well as after anti-inflammatory treatment. However, statistical analyses revealed a strong relationship between the level of PTX3 and disease activity both before (*p* < 0.005, r = 0.63) and after (*p* < 0.05, r = 0.60) treatment. A similar correlation was identified in the case of S100A12 (*p* < 0.005, r = 0.81) but only in the post-treatment group of patients with CD.

Moreover, as the median CRP concentration in the blood of patients with Crohn’s disease was increased compared to the reference values, we estimated the correlation between the level of this inflammatory marker and serum profiles of pro-GN, PTX3 and S100A12. Unfortunately, statistical analyses revealed no significant relationship between the concentration of the analyzed biomarkers and CRP in the pre- and post-treatment groups of patients with CD.

### 3.4. The Effect of Anti-Inflammatory Treatment on the Serum Profiles of Pro-GN, PTX3 and S100A12 in Patients with Crohn’s Disease

**Figure 4:**
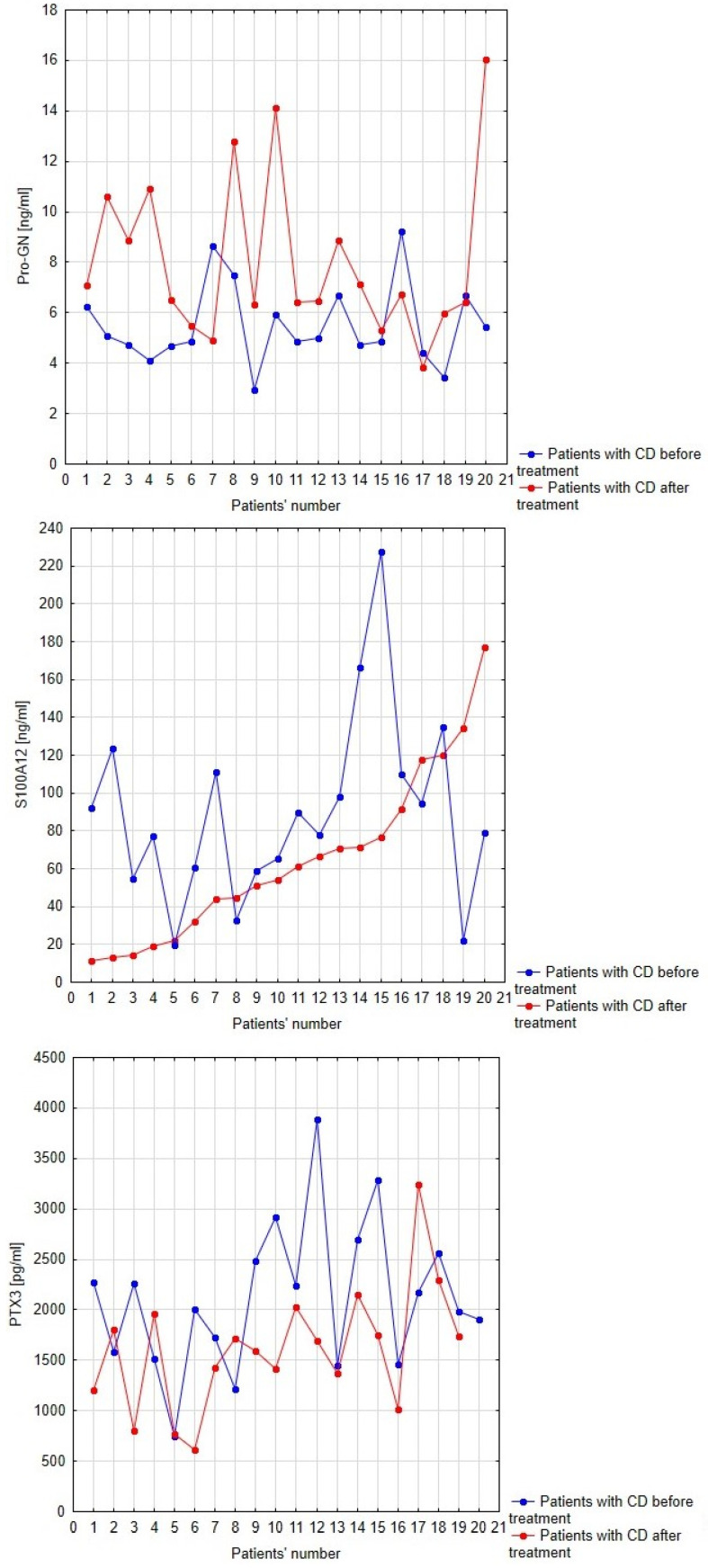
Serum profiles of pro-GN, S100A12 and PTX3 in patients with Crohn’s disease before and after anti-inflammatory treatment with regard to individual patients.

Considering that anti-inflammatory treatment reduced significantly the activity of the disease, we analyzed the impact of therapy on the serum profiles of pro-GN, PTX3 and S100A12 in patients with CD. Serum profiles of pro-GN, S100A12 and PTX3 in patients with Crohn’s disease before and after anti-inflammatory treatment with regard to individual patients is presented in Figure 4. Anti-inflammatory treatment affected significantly the level of pro-GN (*p* < 0.001) and PTX3 (*p* < 0.05). The increase in serum pro-GN concentration reached 46%; however, in comparison to healthy individuals, the level of pro-GN remained decreased (*p* < 0.005). After one year of anti-inflammatory treatment, we observed a decrease in PTX3 levels by 24%, reaching values similar to the control group. However, no significant change was observed in the case of S100A12 after therapy. The level of S100A12 in the post-treatment group of patients with CD remained significantly increased compared to healthy individuals (Figure 4).

### 3.5. Comparison of Serum Profiles of Pro-GN, PTX3 and S100A12 in Patients with Crohn’s Disease and Ulcerative Colitis

In this study, we aimed to evaluate the use of serum pro-GN, PTX3 and S100A12 measurements in the differential diagnosis of inflammatory bowel disease. To achieve this, we referred to our previous research, conducted in the Department of Clinical Chemistry and Laboratory Diagnostics [17], in which we assessed serum pro-GN, PTX3 and S100A12 concentrations in another type of inflammatory bowel disease—ulcerative colitis (UC). The serum profiles of pro-GN, PTX3 and S100A12 in patients with UC are presented in Table 2. Statistical analyses revealed a significant difference in PTX3 and S100A12 levels between patients with CD and UC, but no similar change was observed in case of pro-GN values. Patients with Crohn’s disease presented a decreased concentration of PTX3 (*p* < 0.005), while the level of S100A12 (*p* < 0.05) was increased compared to the subjects with ulcerative colitis before treatment. On the other hand, after implemented treatment, the serum profiles of pro-GN, PTX3 and S100A12 did not differ significantly between UC and CD patients.

## 4. Discussion

### 4.1. Serum Profiles of Pro-GN, PTX3 and S100A12 in Patients with Crohn’s Disease and Healthy Individuals

The pathogenesis of Crohn’s disease is linked to disrupted intestinal barrier integrity, resulting in the activation of the immune response and the subsequent initiation and development of inflammatory process within the intestines [1]. As impaired intestinal barrier integrity contributes to the development of the disease, we assessed the concentration of serum proguanylin, which represents the inactive and most abundant form of GC-C agonist in circulation, in patients with Crohn’s disease and healthy individuals. In the group of patients with CD, we noted a significant decrease in pro-GN level compared to the control group. Obtained results are in line with the study by Volkmann et al. [18], where the level of pro-GN in plasma of patients with Crohn’s disease was also found to be reduced. The mentioned researchers not only noted the same difference between the study and control groups but also obtained comparable results of circulating proguanylin profiles in patients with CD (5.5 ± 1.6 ng/mL in our study vs. 5.1 ± 1.6 ng/mL in Volkmann’s study). Furthermore, the disruption of the guanylin–GC-C pathway in IBD may be confirmed by the research of Brenna et al. [19], in which decreased expression of guanylin, together with reduced GC-C signaling, was noted in biopsies collected from patients with IBD. The observed decrease in pro-GN levels during CD and consequent downregulation of the GC-C signaling pathway may lead to a reduction in the number of goblet cells, resulting in diminished protective mucus production for the intestinal epithelium. Moreover, in the study conducted by Lin et al. [8], the loss of GC-C signaling in vivo was associated with the reduced expression of tight junction proteins, such as occludin, claudin-2 and -4 and junction adhesion molecule A, ultimately leading to increased permeability of the intestinal barrier. Conversely, in a mouse model of dextran sulfate sodium (DSS)-induced colitis, activation of GC-C signaling by guanylin prevented from the disruption of intestinal barrier integrity [8]. These findings collectively indicate that decreased levels of proguanylin and guanylin may contribute to impaired intestinal barrier integrity, representing a key factor in the pathogenesis of Crohn’s disease. Given the significant difference in the serum profile of pro-GN between patients with Crohn’s disease and healthy individuals, the mentioned biomarker shows potential for use in the diagnosis of the disease.

In this study, we also assessed the concentration of pentraxin 3 and S100A12, which are proteins related to the innate immune response and are engaged in the development of inflammation, being an integral part of the pathogenic process during Crohn’s disease. The conducted statistical analyses revealed a significant difference in the concentration of both PTX3 and S100A12 between patients with CD and healthy individuals. The levels of PTX3 and S100A12 in the serum of CD patients were markedly increased. A similar increase in PTX3 expression was also reported in the study performed by Chen et al. [20], where PTX3 was found to be elevated in the serum and biopsies of inflamed intestinal tissue collected from patients with CD. The observed increase in PTX3 concentration in the serum of CD patients is probably related to the ongoing inflammatory state, as it functions as an acute phase protein. In the early phase of Crohn’s disease, neutrophils migrate to the inflammatory site, where they can form NETs to immobilize pathogens penetrating the intestinal barrier. The formation of NETs is accompanied by the degranulation of neutrophils granules, leading to the release of various components, including PTX3. Moreover, the enhanced release of pro-inflammatory cytokines like TNF-α can stimulate the synthesis and secretion of PTX3 from another cellular source—macrophages [9]. A similar observation can be made in the case of S100A12 protein; its increase in the serum of CD patients was confirmed in the study by Foell et al. [21]. Similar to PTX3, the primary cellular sources of S100A12 are neutrophils, although it can be secreted also by macrophages after exposition to TNF-α or/and IL-6. In the course of CD, after release from cells, S100A12 can induce the RAGE-mediated activation of the NF-κB pathway, resulting in the synthesis of pro-inflammatory cytokines and adhesion molecules. The pro-inflammatory properties of S100A12 may lead to a vicious cycle of inflammation, in which intensive activation of NF-κB leads to the release of S100A12 and RAGE-mediated upregulation of NF-κB, further intensifying inflammatory process across intestinal mucosa [12]. Considering the role of the analyzed proteins in the pathogenic process and the obtained results, the measurements of serum PTX3 and S100A12 may be used during the diagnostic process for Crohn’s disease.

### 4.2. The Relationship between Serum Levels of Pro-GN, PTX3 and S100A12 and Disease Activity in Patients with Crohn’s Disease

Regarding the crucial relationship between the analyzed biomarkers and the pathogenic process during Crohn’s disease, we also assessed the usefulness of serum pro-GN, PTX3 and S100A12 measurements in evaluating disease activity. The conducted statistical analyses revealed no significant relationship between pro-GN concentration and the CDAI score. However, we noted a significant correlation between the profile of PTX3 and disease activity both before and after anti-inflammatory treatment. Corresponding results were presented in Chen et al.’s study [20], where a statistically significant association between serum PTX3 level and disease activity, expressed by CDAI score, was found in the CD patients group. Given the inflammatory basis of the disease, the observed positive and strong correlation may be related to the fact that pentraxin 3 is an acute phase protein, leading to an increase in concentration corresponding to spreading inflammation across the intestines. Moreover, as neutrophils serve as a reservoir of the mentioned protein, the increase in the PTX3 level may be associated with an intensified influx of these cells to lesions. The activation of neutrophils and subsequent NET formation may play a pathogenic role, especially when accumulated. Upon degranulation of their granules, reactive oxygen species (ROS) and proteases like matrix metalloproteinase or neutrophil elastase are released, which can further promote tissue damage and inflammation, thereby enhancing the disease activity [2]. Another cause of increased PTX3 concentration in the serum of patients with CD might be its persistent secretion in response to adequate stimuli. One of the stimuli enhancing the synthesis and release of PTX3 is TNF-α; this cytokine is elevated during CD [10,22]. In the research conducted by Reimund et al. [22], an increased level of TNF-α was noted in the supernatant of inflamed intestinal biopsies collected from CD patients compared to the non-IBD control. At the same time, the concentration of TNF-α in the supernatant of uninflamed intestinal biopsies was elevated compared to controls, although it was lower than the supernatant of inflamed intestinal biopsies. These results highlight the critical role of TNF-α in the initiation and progression of the disease but also may indicate a potential cause of PTX3 upregulation during the active phase of disease. Consequently, PTX3 measurements in serum may reflect the intensity of the inflammatory state, being a key process in pathogenesis and development of Crohn’s disease.

Another significant correlation in this study was found between the level of S100A12 protein and the CDAI score in the group of patients with CD, but only after anti-inflammatory treatment. Similar to the results obtained in our study, Däbritz et al. [23] also noted a significant relationship between the CDAI score and the concentration of S100A12 protein in serum and feces of patients with CD. The observed correlation, as in the case of PTX3, may be a result of enhanced influx of neutrophils during active disease as well as the increased level of TNF-α inducing the synthesis of S100A12 in macrophages. Moreover, S100A12 may play a causative role in the progression of the disease, as it can enhance the inflammatory response through the S100A12–RAGE–NF-κB axis, thereby creating a vicious cycle of inflammation. Additionally, the RAGE-mediated activation of NF-κB may have a prolonged character, as upregulation of the mentioned pathway by a complex of advanced glycation products (AGE) and RAGE lasts longer compared to agonists like TNF-α binding to non-RAGE receptors [24]. While this character of NF-κB activation is not confirmed in the case of the S100A12–RAGE complex, it may explain the relationship between activity of the disease and the S100A12 level in circulation of CD patients. The lack of a statistically significant relationship between S100A12 serum level and disease activity in the pretreatment group of CD patients may be related to diverse disease locations and characters of the disease in patients before treatment. In Däbritz et al.’s study [23], the S100A12 serum profile differed according to location of the disease, with patients with isolated colonic disease presenting an increased concentration of circulating S100A12 compared to patients with ileal or ileocolonic disease. Moreover, a valid difference was also noted in the case of the character of the disease. The highest level of S100A12 was observed in patients with penetrating disease compared to those with stricturing disease [23]. Regarding the significant relationship between PTX3, S100A12 and disease activity, these mentioned biomarkers might be useful in monitoring the disease activity. Considering the significant correlation between PTX3 levels and CDAI scores both before and after treatment, in contrast to the case of S100A12 where similar observation was made only in the post-treatment group, it can be assumed that PTX3 is a biomarker that better reflects the disease activity in CD.

### 4.3. The Effect of Anti-Inflammatory Treatment on the Serum Profiles of Pro-GN, PTX3 and S100A12 in Patients with Crohn’s Disease

In this study, we analyzed the levels of pro-GN, PTX3 and S100A12 in the serum of patients before and after one year of anti-inflammatory therapy. Implemented treatment significantly decreased the activity of the disease in patients with CD. Moreover, in the post-treatment group of patients with CD, we noted a significant increase in the pro-GN level in circulation; however, compared to the control group, the pro-GN concentration remained decreased. These results can be explained by the study conducted by Brenna et al. [19], where the expression of guanylin was found to negatively correlate with the expression of pro-inflammatory cytokines (TNF-α, IL-1β) in inflamed biopsies collected from patients with IBD. Therefore, one year of anti-inflammatory treatment resulting in the amelioration of the inflammatory process across intestinal mucosa might be related to enhanced synthesis of proguanylin and its release from IECs. These findings suggest that measurements of pro-GN may hold potential for assessing the proper response to anti-inflammatory treatment during CD.

In group of patients with Crohn’s disease, the implemented anti-inflammatory treatment significantly decreased the concentration of PTX3, reaching levels similar to those measured in the serum of healthy individuals. As pro-inflammatory cytokines stimulate the synthesis and release of PTX3 from neutrophils and macrophages, the observed decline in PTX3 levels may reflect the diminishing of the pro-inflammatory state across the intestinal mucosa [9,10]. Furthermore, considering the previously noted strong and positive correlation between the serum profile of PTX3 and CDAI score, the observed decrease in this biomarker may indicate the reduction in disease activity after anti-inflammatory treatment. Therefore, the measurements of the serum profile of PTX3 might be a helpful tool in monitoring the effect of implemented treatment in Crohn’s disease. Meanwhile, the level of S100A12 also decreased, however not statistically significantly, indicating the predominance of pro-GN and PTX3 assessments in evaluating the beneficial effect of corticosteroid therapy in the course of Crohn’s disease. These observations should, however, be further explored at different time intervals since in our study, measurements of the proposed biomarkers were only conducted after one year of treatment. More frequent sample collection and biochemical analyses could provide valuable insights into clinical fluctuations observed during treatment.

### 4.4. Comparison of Serum Profiles of Pro-GN, PTX3 and S100A12 in Patients with Crohn’s Disease and Ulcerative Colitis

In our previous study, we assessed the serum profiles of pro-GN, PTX3 and S100A12 protein in patients with ulcerative colitis [17]. In this study, we aimed to compare the concentrations of these mentioned biomarkers between patients with UC and CD to evaluate their usefulness in the differential diagnosis of IBD. The conducted statistical analysis revealed no difference in the profile of pro-GN; however, the levels of PTX3 and S100A12 differed significantly among the analyzed pretreatment groups. Although some of the stimuli enhancing the secretion of PTX3 and S100A12 were the same, including TNF-α, the serum profiles of these proteins differed between CD and UC patients [10,21]. Specifically, the concentration of PTX3 was increased in the group of patients with ulcerative colitis, while the S100A12 level was elevated in patients with Crohn’s disease. The observed differences might be related to differences in the immunological patterns of the diseases, specifying varied activity of immune cells and cytokine profile [25,26].

The increase in PTX3 level in the serum of patients with ulcerative colitis compared to those with Crohn’s disease may be related to enhanced NET formation during UC, as noted in the study conducted by Dinallo et al. [27]. The mentioned researchers assessed the expression of protein arginine deiminase-4 (PAD4) leading to the formation of NETs in biopsies collected from patients with CD and UC. Enhanced expression of PAD4 was noted in UC patients compared to CD or healthy individuals, while no significant difference in PAD4 expression was observed between samples collected from the CD and control groups [27]. These results indicate that NET formation is enhanced during UC in comparison to CD. Consequently, the increase in PTX3 level in the serum of patients with UC may be a result of enhanced formation of NETs, during which components of neutrophils granules including PTX3 are released. Further confirmation of differences in NET formation between UC and CD can be found in the study conducted by Angelidou et al. [28]. This study not only confirmed the results obtained by Dinallo et al. but also indicated that the expression of active IL-1β is increased in NETs isolated from biopsies of UC patients compared to CD. IL-1β is one of the factors enhancing the synthesis of PTX3; therefore its elevated expression in NETs may result in upregulation of PTX3 during ulcerative colitis [28]. These presented results provide evidence that PTX3 measurements can be useful in differentiating Crohn’s disease from ulcerative colitis during the diagnostic process.

Among patients with Crohn’s disease and ulcerative colitis, another difference was noted in the case of S100A12, the concentration of which was increased in the serum of CD patients compared to UC. These results may be further confirmed by the Foell et al.’s [21] study, in which an elevated level of S100A12 was observed in the serum of CD patients compared to patients with UC. Considering that one of the cellular sources of S100A12, similar to PTX3, are neutrophils and enhanced NET formation was noted in the case of UC patients in contrast to CD, it could expected that S100A12 levels would be increased in UC patients as well. However, as observed in our study and Foell’s, the increase in S100A12 protein in CD may be related to the fact that S100A12 is a cytosolic protein and it can be secreted without prior degranulation of the neutrophils’ granules or NET formation. Another explanation for the increased S100A12 level during CD might be the release of this protein from a different cellular source—macrophages. One of the factors enhancing the synthesis and release of S100A12 from macrophages is IL-6 [11,29]. In the study by Hasegawa et al. [29], IL-6 was found to increase the level of S100A12 mRNA in a dose- and time-dependent manner as well as to enhance the release of S100A12 protein from macrophages. Despite the fact that alterations in activity of dendritic cells (DCs) are related to abnormal immune response to pathogens penetrating the IECs in both CD and UC, colonic DCs present crucial phenotype differences according to the type of the disease. In the study by Hart [30] in which biopsies were collected from patients with UC and CD, the number of colonic IL-6-producing DCs was assessed using intracellular staining. Researchers revealed that patients with CD presented an increased number of DCs synthesizing IL-6 compared to patients with UC and healthy individuals, while no significant difference in the number of these cells was noted between the UC and control groups. These results indicate that colonic DCs synthesize and release greater amounts of IL-6 in Crohn’s disease than in ulcerative colitis, which consequently may lead to enhanced expression of S100A12 in macrophages during CD. It needs to be taken into consideration that divergent types of CD might be associated with differences in the serum profile of the analyzed biomarkers. In our research, the location of the disease was inconsistent in patients with CD; therefore, considering the insufficient test power in the L1–L4 subgroups, we could not assess the utility of these biomarkers in distinguishing the ileal, colonic and ileocolonic CD. However, regarding the crucial differences in PTX3 and S100A12 serum profiles in patients with Crohn’s disease and ulcerative colitis, together with its varied synthesis and release during the diseases, our study indicate that these biomarkers might be useful in the differential diagnosis of IBD.

## 5. Conclusions

Regarding the divergent phenotypes of Crohn’s disease and its possible extraintestinal manifestation, the diagnosis of the disease remains a significant challenge for clinicists. Therefore, the aim of our study was to identify new possible biomarkers of Crohn’s disease that could support the diagnostic process and disease monitoring. In our research, we assessed the concentration of a biomarker related to intestinal barrier integrity, proguanylin, and proteins related to inflammatory process, namely, pentraxin 3 and S100A12. The conducted statistical analyses revealed a significant difference in the serum profiles of pro-GN, PTX3 and S100A12 between patients with Crohn’s disease and healthy individuals, indicating the diagnostic usefulness of all the analyzed biomarkers in this disease. Moreover, a crucial relationship was noted between the level of PTX3 and the CDAI score, which assesses the disease activity, both before and after anti-inflammatory treatment. A similar correlation was observed for S100A12 serum concentration and disease activity but only in the post-treatment group of patients. These findings provide evidence that PTX3 and S100A12 measurements might be helpful in evaluating disease activity. Furthermore, we also estimated the effect of the implemented anti-inflammatory treatment based on prednisone on the profile of analyzed biomarkers and noted a significant difference in pro-GN and PTX3 levels, confirming the potential use of these parameters in evaluating the proper response to treatment. As in our previously conducted study, we assessed the levels of pro-GN, PTX3 and S100A12 in serum of patients with ulcerative colitis, and we also compared the profiles of these biomarkers among patients with CD and UC. We revealed a significant difference in the serum concentration of PTX3 and S100A12 protein between patients with CD and UC, highlighting the usefulness of these biomarkers in the differential diagnosis of IBD. However, the presented results should be interpreted with caution and confirmed on the greater study group. The results of our study not only provide new insights into the molecular basis of Chron’s disease but also can be helpful in the diagnosis and monitoring the disease’s activity.

## Figures and Tables

**Table 1:** Clinical characteristics of patients with Crohn’s disease.

Parameter	CD0	CD1	*p*
Age (years)	32.1 ± 9.32		
CDAI score	303.4 ± 52.45	270.81 ± 44.30	0.024
CRP (mg/L)	15.7 (4.2–39.05)	15.2 (5.3–23.40)	0.242
Sodium (mmol/L)	138.15 ± 2.89	138.38 ± 3.9	0.886
Potassium (mmol/L)	4.35 (4.2–4.45)	4.35 (4.20–4.45)	0.606
Glucose (mmol/L)	5.11 ± 0.86	4.89 ± 0.43	0.205
Creatinine (μmol/L)	81.33 ± 14.14	86.63 ± 13.26	0.300
WBC (×10^3^/L)	7.2 ± 3.21	6.22 ± 1.97	0.630
RBC (×10^6^/L)	4.04 (3.78–4.71)	4.16(3.82–4.86)	0.803
Hemoglobin (g/dL)	11.58 ±2.3	12.41 ± 1.94	0.411
HCT (%)	35.04 ± 5.54	37.09 ± 5.1	0.405
MCV (fL)	85.81 ± 8.44	86.21 ± 6.65	0.908
MCH (pg)	29.30 (26.2–31.45)	29.7 (27.65–30.5)	0.803
MCHC (g/dL)	33.05 (31.45–34)	33.15 (32.35–34.00)	0.803
RDWCV (%)	15.14 ± 2.35	14.01 ± 1.48	0.171
PLT (×10^9^/L)	349 (263–400)	253 (169.5–323.5)	0.047
PCT (%)	0.34 (0.26–0.37)	0.27 (0.18–0.32)	0.108
PLCR (%)	20.73 ± 5.23	26.73 ± 9.70	0.030
PDW (fL)	9.80 (9.40–10.90)	11.55 (9.75–12.65)	0.041
MPV (fL)	9.43 ± 0.72	10.28 ± 1.30	0.020

Comparison of selected laboratory parameters before and after a year of anti-inflammatory treatment. Data are presented as mean ± standard deviation (SD) in normally distributed data or median and interquartile range (25th–75th percentile) in not normally distributed data. Data were analyzed using Student’s t test or sign test. *p* < 0.05 is statistically significant. CD, Crohn’s disease; CD0, patients with Crohn’s disease before anti-inflammatory treatment; CD1, patients with Crohn’s disease after a year of anti-inflammatory treatment; CDAI, Crohn’s Disease Activity Index; CRP, C-reactive protein; HCT, hematocrit; MCH, mean corpuscular hemoglobin; MCHC, mean corpuscular hemoglobin concentration; MCV, mean corpuscular volume; MPV, mean platelet volume; PLCR, platelet–large cell ratio; PLT, platelet count; PCT, platelecrit; RBC, red blood cells; RDWCV, red blood cell distribution width, coefficient of variation; WBC, white blood cells.

**Table 2:** Serum profile of pro-GN, S100A12 and PTX3 in healthy individuals, patients with Crohn’s disease and patients with ulcerative colitis.

Parameter	C	CD0	#UC0	*p* CD0 vs. UC0
Age (years)	37.95 ± 10.42	32.1 ± 9.32	33.38 ± 12.75	
Pro-GN (ng/mL)	11.35 ± 2.59	5.5 ± 1.6	#5.8 ± 2.8	*p* > 0.05
PTX3 (pg/mL)	1608.37 ± 587.05	2117.9 ± 737.4	#3502.1 ± 1881.5	*p* < 0.005
S100A12 (ng/mL)	19.74 ± 8.07	79.4 ± 39.5	#57.4 ± 46.6	*p* < 0.05

Results are presented as mean ± standard deviation in normally distributed data. Levels of pro-GN, PTX3 and S100A12 in the serum of patients with ulcerative colitis were measured in our previous research [17]. #, results obtained in our previous research [17]; C, control group; CD0, patients with Crohn’s disease before anti-inflammatory treatment; pro-GN, proguanylin; PTX3, pentraxin 3; UC0, patients with ulcerative colitis before biological treatment with adalimumab.

**Table 3:** Relationship between the concentration of pro-GN, S100A12 and PTX3 with disease activity expressed as CDAI index in patients with Crohn’s disease.

Parameter	Disease Activity Index (CDAI)	
	Crohn’s Disease Patients before One Year of Anti- Inflammatory Treatment (CD0)	Crohn’s Disease Patients after One Year of Anti- Inflammatory Treatment (CD1)
Pro-GN (ng/mL)	r= −0.32; *p* > 0.05	r= 0.12; *p* > 0.05
PTX3 (pg/mL)	**r = 0.63; *p* < 0.005**	**r = 0.60; *p* < 0.05**
S100A12 (ng/mL)	r= 0.33; *p* > 0.05	**r = 0.81; *p* < 0.005**

Pearson correlation coefficients between the concentration of analyzed biomarker and disease activity with statistical significance is bolded. CDAI, Crohn’s Disease Activity Index.

## Data Availability

Data are contained within the article.
